# Multiple retinal astrocytic hamartomas in siblings with lebers congenital amaurosis: a case series and review of literature

**DOI:** 10.1186/s12886-020-01646-z

**Published:** 2020-09-23

**Authors:** Lagan Paul, Sumit Kumar, Shalini Singh, Tanya Jain

**Affiliations:** Department of Vitreo-Retina Services, Dr. Shroff Charity Eye Hospital, 5027, Kedarnath Lane, Daryaganj, Delhi, New Delhi 110002 India

**Keywords:** Case report, Lebers congenital amaurosis (LCA), Retinal astrocytic hamartomas (RAHs), Inherited retinal disorder (IRD)

## Abstract

**Background:**

Leber’s congenital amaurosis (LCA) is a known inherited retinal disease (IRD) associated with severe visual loss, nystagmus, amaurotic pupils, oculo-digital sign and markedly reduced or absent electroretinograms (ERG). Retinal astrocytic hamartomas (RAH) is a benign vascularized glial tumor of the retina. There is no known association of these two entities, more so in siblings.

**Case presentation:**

A pair of siblings diagnosed as LCA who presented with RAH with no extraocular symptoms or signs of phakomatosis were imaged. Multimodal imaging was performed and are elaborately described in this article.

**Conclusion:**

LCA in siblings with multiple RAHs is an extremely rare association. Recent advances in retinal imaging tools have aided in diagnosing even subtle and early RAH with high sensitivity using Infrared imaging (IRI) and Optical coherence tomography (OCT).

## Background

Lebers Congenital Amaurosis (LCA) is an inherited retinal disorder (IRD) which is characterized by severe visual loss which maybe present congenitally or in early infancy. It is associated with nystagmus, amaurotic pupils, oculo-digital sign and markedly reduced or absent electroretinograms (ERG) [1]. We report two siblings with non-syndromic forms of LCA with multiple retinal astrocytic hamartomas (RAHs) in the eyes. We describe the findings of multimodal imaging techniques in these eyes in the present case report.

## Case presentation

**Fig. 1:**
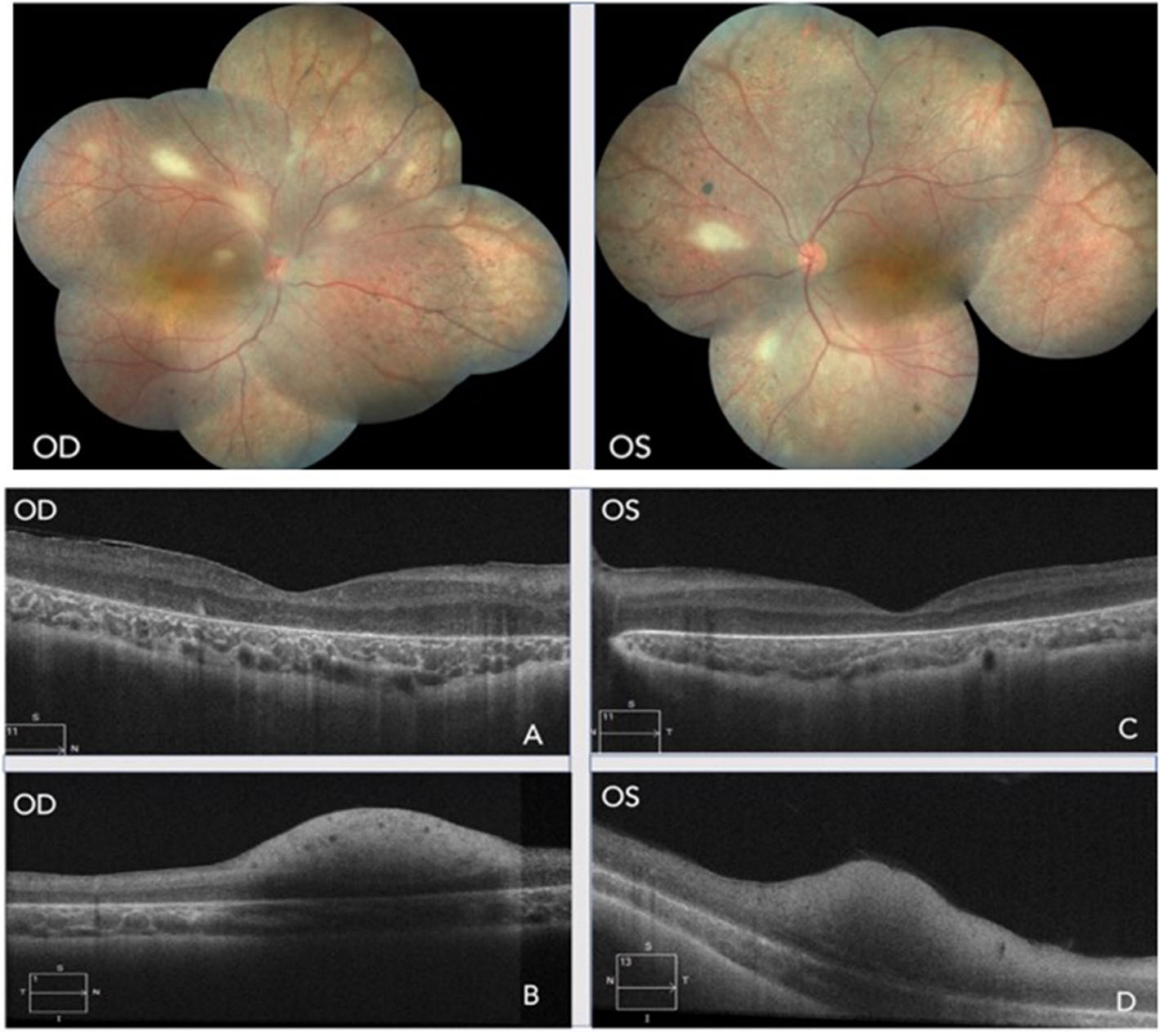
Colored fundus picture of RE and LE showing generalized vascular attenuation, RPE alterations and multiple RAH. **a** SD-OCT of RE through fovea showing altered IS-OS junction. **b** SD-OCT of RE through the lesion showing hyper-reflective lesion in nerve fiber layer with posterior shadowing without any calcifications. **c**- SD-OCT of LE through fovea showing altered IS-OS junction. **d** SD-OCT of LE through the lesion showing hyper-reflective lesion in nerve fiber layer with posterior shadowing without any calcifications

An 8 years old boy presented with complaint of painless visual deterioration in both eyes (BE) since early childhood. On examination, the best corrected visual acuity (BCVA) in the right eye (RE) was 6/18, N18 and in the left eye (LE) was counting finger at 10 cm, N60 with nystagmus in BE. Supero-nasal subluxation of the lens was present in BE. Applanation tonometry was normal in BE. Fundus examination of BE showed normal optic disc with generalized attenuation of vessels with retinal pigment epithelium (RPE) atrophy and pigmentary alterations in the retina. A reddish-orange hue was seen at the fovea and atrophic maculopathy was noted. There were characteristic multiple off -white relatively well defined lesions, which were more marked along the arcades in the RE with a single lesion located on the inferonasal retinal quadrant in the fellow eye (Fig. 1).

Spectral domain optical coherence tomography (SD-OCT) depicted sub-foveal thinning with RPE atrophy in the RE (Fig. 1a). Scans passing just over the lesion revealed a hyperreflective dome shaped mass within the nerve fiber layer (NFL) along with a posterior optical shadowing associated with the disorganization of inner retinal layers (Fig. 1b). SD-OCT scans of the fellow eye revealed similar features in the LE (Fig. 1c and d).

**Fig. 2:**
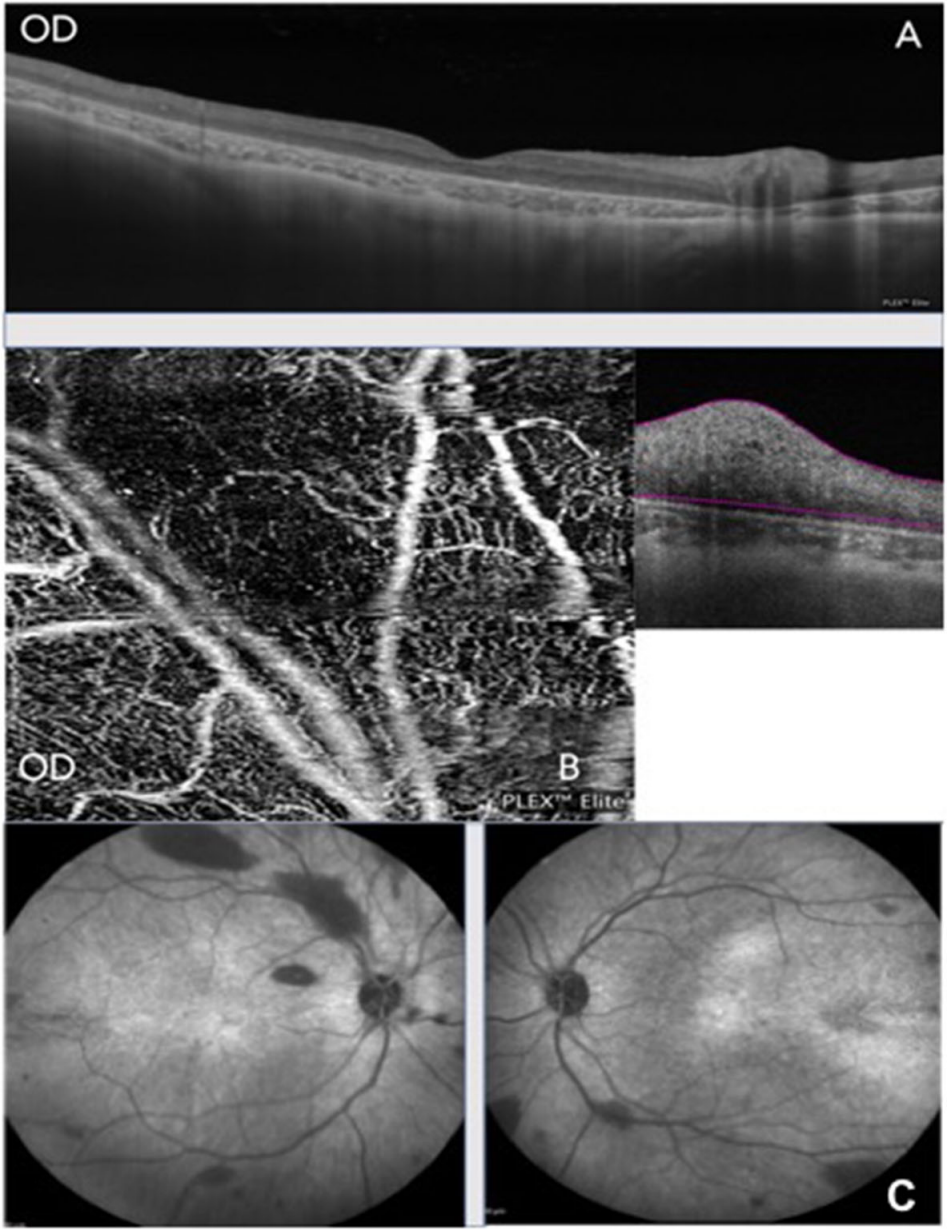
**a** SS-OCT of RE showing thinned out retina and choroid. **b** OCT-A of RE through the lesion showing abnormal capillary network. **c**- IRI of RE and LE showing multiple hypo-reflectance lesions

**Fig. 3:**
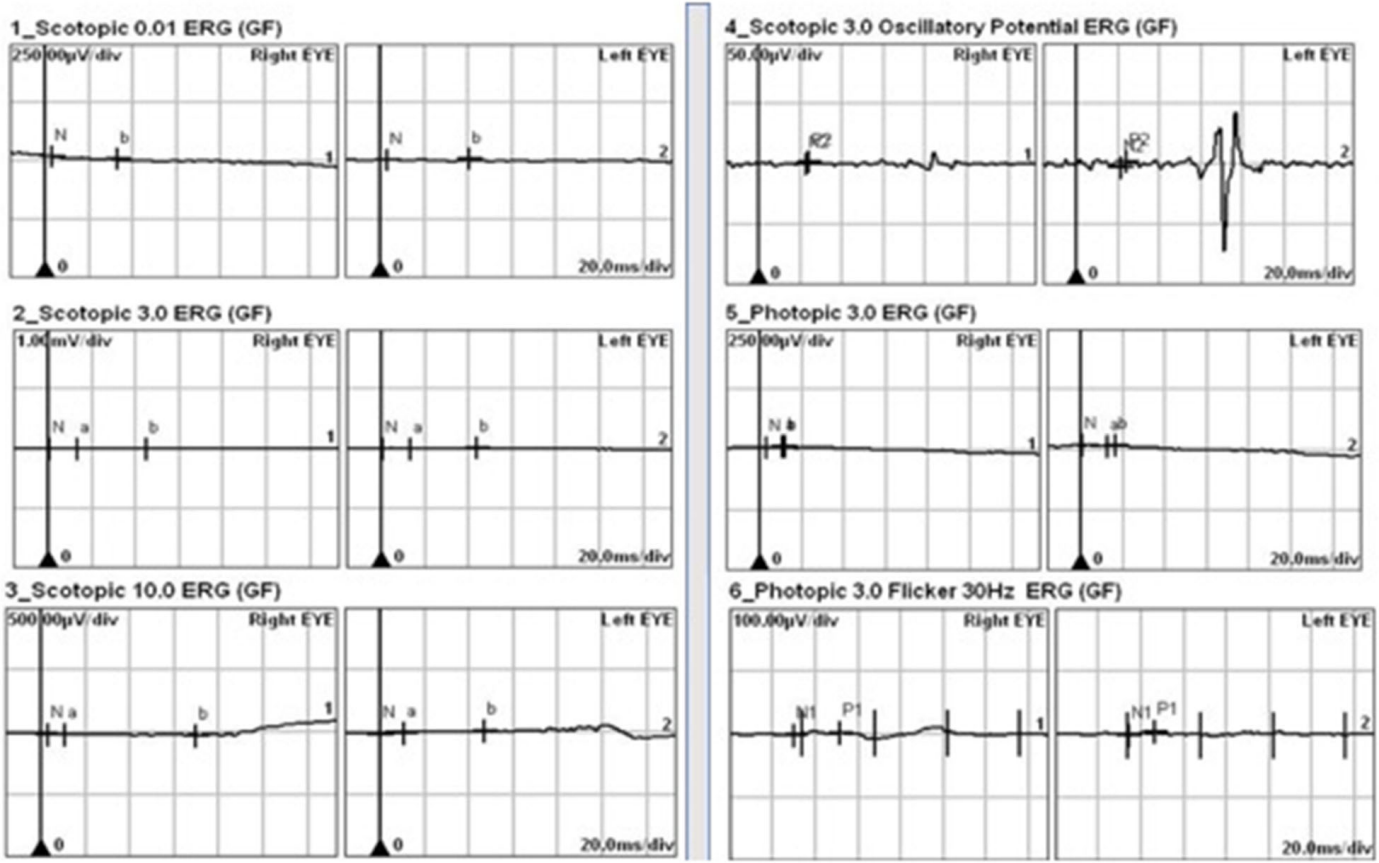
ERG showing grossly diminished photopic and scotopic response

SS-OCT (Swept-Source Optical Coherence tomography) through the fovea of RE showed an opaque lesion arising in the NFL with a smooth transition from retina and posterior shadowing. All other retinal layers appeared compressed along with mild thinning of choroid beneath the tumorous lesion (Fig. 2a). However, SS-OCT of LE could not be performed due to nystagmus and poor fixation. OCT-A over the retinal tumor showed a feeder vessel with an abnormal network of capillary plexus emanating from it (Fig. 2b). Autofluorescence (AF) was inconclusive in BE. Infra-red imaging (IRI) showed numerous hypo reflectance lesions in BE corresponding to RAH at the posterior pole (Fig. 2c). ERG showed markedly extinguished responses with both photopic and scotopic stimuli (Fig. 3).

Systemic evaluation by a pediatrician revealed no signs of Tuberous sclerosis or any associated syndrome. There were no systemic features of renal or hepatic dysfunction, neurological deficit, phacomatoses or deafness. MRI (Magnetic resonance imaging) cranium and abdominal ultrasound was normal along with normal renal functions. Gene mapping could not be performed due to limited financial resources.

**Fig. 4:**
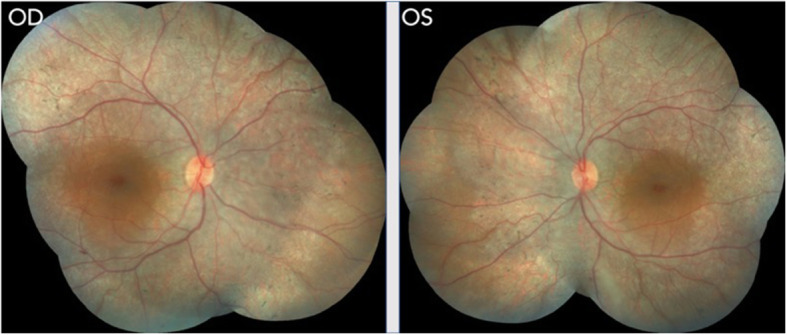
Colored fundus picture of RE and LE of the elder sister showing generalized vascular attenuation and RPE alterations

His pedigree charting revealed a high refractive error in his sister. She was also assessed for any associated syndromes. She was 11 years old, and her BCVA was 6/9, N6 with normal slit-lamp biomicroscopic findings in both eyes. Fundus evaluation revealed some pigmentary alteration in BE with normal macula (Fig. 4). An isolated retinal hamartoma was noted in far periphery in the RE.

Based on the above clinical findings of severe visual loss, nystagmus, hyperopia, amaurotic pupils, abnormally pigmented fundi and macular atrophy and with detailed systemic investigations, a diagnosis of presumed non- syndromic LCA with RAH was made. Since multiple alleles have been associated with LCA, gene therapy is highly costly [2] and since RAHs are known to have a benign natural course [2], we preferred to observe them with a follow-up every 6 months.

## Discussion and conclusion

The prevalence of LCA is between 1 in 33,000 to 1 in 81,000 and accounts for ≥5% of all IRD [3, 4]. It is characterized by severely impaired vision from birth or first few months of life, and often accompanied by nystagmus and poor pupillary light responses The retina may be normal looking or develop abnormalities like disc pallor, vascular attenuation, mild peripheral pigmentary retinopathy or atrophic maculopathy [1]. The affected infants usually have high hyperopia with severely affected or undetectable ERG [1]. RAH is a benign vascularized glial tumor of the retina which could be acquired or congenital, wherein congenital RAH are more common and strongly associated with tuberous sclerosis complex.

Ambiya et al. [5] reported an optic nerve head hamartoma with LCA. However, to the best of our knowledge and the literature available, we report the first case of two siblings with non-syndromic LCA with RAHs. The male child showed multiple hamartoma scattered throughout the fundus with poor visual acuity, while his elder sister showed a single retinal hamartoma in far periphery in the right eye and maintained a good vision in both eyes.

Recently, studies on RAHs have highlighted the role of IRI and SD-OCT in detecting subtle changes which were otherwise not visible on clinical examination or colored fundus photography or AF [6, 7] which make them indispensable tool in detecting subtle or small tumors and following up these eyes. IRI in our patient reflected > 14 and 9 hamartomas in RE and LE while RFI could highlight only 7 and 2 in RE and LE respectively. We postulate that IRI could be used to monitor the other sibling for development of any acquired hamartomas in the future. The usability and repeatability of SS-OCT and OCT-A are identical to the description in the available literature [8, 9].

In conclusion, we describe siblings with clinical and ERG findings suggestive of LCA associated with RAHs. The RAHs appear as hyper-reflective dome shaped lesions at the level of NFL and reveal a feeder vessel on the OCT-A scans. We furthermore highlight the role of IRI in detecting subtle changes which are otherwise not detectable on fundus examination and monitoring these patients.

## Data Availability

N/A

## Abbreviations

LCA: Lebers Congenital Amaurosis
IRD: Inherited retinal disorder
ERG: Electroretinograms
RAH: Retinal astrocytic hamartoma
BCVA: Best corrected visual acuity
RPE: Retinal pigment epithelium
SD-OCT: Spectral domain – Optical coherence tomography
NFL: Nerve fiber layer
IRI: Infra-red imaging
OCT-A: Optical coherence tomography- angiography

## References

[1] Agarwal A, Gass J (2012). Gass' atlas of macular diseases.

[2] Leber’s congenital amaurosis and the role of gene therapy in congenital retinal disorders. Int J Ophthalmol. 2017. 10.18240/ijo.2017.03.24 PMC5360787 28393043

[3] Koenekoop R (2004). An overview of leber congenital amaurosis: a model to understand human retinal development. Surv Ophthalmol.

[4] Stone E (2007). Leber Congenital Amaurosis–A Model for Efficient Genetic Testing of Heterogeneous Disorders: LXIV Edward Jackson Memorial Lecture. Am J Ophthalmol.

[5] Ambiya V, Kuppermann B, Narayanan R. Retinal astrocytic hamartoma in a patient with Leber's congenital amaurosis. Case Reports. 2015;2015(mar03 1):bcr2014208374-bcr2014208374. 10.1136/bcr-2014-208374 PMC4369010 25737223

[6] Say EAT, Shah SU, Ferenczy S, Shields CL. Optical coherence tomography of retinal and choroidal tumours. J Ophthalmol. 2011. 10.1155/2011/385058 PMC3145171 21811667

[7] Xu L, Burke T, Greenberg J, Mahajan V, Tsang S (2012). Infrared Imaging and Optical Coherence Tomography Reveal Early-Stage Astrocytic Hamartomas Not Detectable by Fundoscopy. Am J Ophthalmol.

[8] Despréaux R, Mrejen S, Quentel G, Cohen S. En Face Optical Coherence Tomography (Oct) And Oct Angiography Findings In Retinal Astrocytic Hamartomas. Retinal Cases Brief Reports. 2016:1. 10.1097/ICB.0000000000000374 27508423

[9] Zimmer-Galler I, Robertson D (1995). Long-term observation of retinal lesions in tuberous sclerosis. Am J Ophthalmol.

